# Establishment and Validation of a Liquid Chromatography-Tandem Mass Spectrometry Method for the Determination of Tigecycline in Critically Ill Patients

**DOI:** 10.1155/2020/6671392

**Published:** 2020-12-29

**Authors:** Fen Yao, Yifan Wang, Yating Hou, Xipei Wang, Jinhua Lan, Zheng Wu, Yirong Wang, Chunbo Chen

**Affiliations:** ^1^School of Biology and Biological Engineering, South China University of Technology, Guangzhou 510006, Guangdong, China; ^2^Department of Emergency Medicine, Maoming People's Hospital, 101 Weimin Road, Maoming 525000, Guangdong, China; ^3^Department of Medical Sciences, Guangdong Provincial People's Hospital, Guangdong Academy of Medical Sciences, Guangdong Cardiovascular Institute, 106 Zhongshan Er Road, Guangzhou 510080, China; ^4^Department of Critical Care Medicine, Guangdong Provincial People's Hospital, Guangdong Academy of Medical Sciences, 106 Zhongshan Er Road, Guangzhou 510080, Guangdong, China; ^5^Department of Intensive Care Unit of Cardiovascular Surgery, Guangdong Cardiovascular Institute, Guangdong Provincial People's Hospital, Guangdong Academy of Medical Sciences, Laboratory of South China Structural Heart Disease, 96 Dongchuan Road, Guangzhou 510080, Guangdong, China; ^6^The Second School of Clinical Medicine, Southern Medical University, Guangzhou, China

## Abstract

Utilizing tigecycline-d9 as an internal standard (IS), we establish and validate a simple, effective, and rapid liquid chromatography-tandem mass spectrometry (LC-MS/MS) method for the quantitative measurement of tigecycline (TGC) in patient plasma. Acetonitrile was used as a precipitant to process plasma samples by a protein precipitation method. The analyte and IS were separated on an HSS T3 (2.1 × 100 mm, 3.5 *μ*m) chromatographic column using isocratic program with a mobile phase comprising of 80% solvent A (water containing 0.1% formic acid (v/v) with 5 mM ammonium acetate) and 20% solvent B (acetonitrile) with a flow rate of 0.3 mL/min. The mass spectrometer, scanning in multireaction monitoring (MRM) mode and using an electrospray ion source (ESI), operated in the positive-ion mode. The ion pairs used for quantitative analysis were *m*/*z* 586.4 ⟶ 513.3 and *m*/*z* 595.5 ⟶ 514.3 for TGC and the IS, respectively. The range of the linear calibration curve obtained with this approach was 50–5000 ng/ml. Intra- and interbatch precision for TGC quantitation were less than 7.2%, and the accuracy ranged from 93.4 to 101.8%. The IS-normalized matrix effect was 87 to 104%. Due to its high precision and accuracy, this novel method allows for fast quantitation of TGC with a total analysis time of 2 min. This approach was effectively applied to study the pharmacokinetics of TGC in critically ill adult patients.

## 1. Introduction

Tigecycline (TGC) is the first member of the glycylcycline class of antimicrobial agents and is associated with refractory infections in critically ill patients [1, 2]. Though high-dose tigecycline (200 mg loading dose, 100 mg q12 h) were recommended for the treatment of severe infections [3–5], a black box warning of increased all-cause mortality was issued by the Food and Drug Administration (FDA) [6]. The area under the concentration-time curve from 0 to 24 h at stead -state (AUC_24_) divided by the minimal inhibitory concentration (MIC) is often used as the pharmacokinetic/pharmacodynamic (PK/PD) index, with target values calculated for various infections [7–11]. Body surface area and creatinine clearance were reported to have a significant influence on clearance (CL) [10, 12, 13]. Renal impairment frequently occurs in critically ill patients [14–18]. Therefore, therapeutic drug monitoring (TDM) of TGC is essential for the optimal use of TGC in clinical practice.

Determinations of TGC in patient plasma by liquid chromatography with ultraviolet (UV) or tandem mass spectrometry (LC-MS/MS) methods were reported previously [19–22]. However, long run times, time-consuming sample preparation, and large sample volumes are needed in these methods [10, 11, 13, 21]. Jiao Xie described an LC-MS/MS method with a short run time of 5 min but a relatively large sample volume of 200 *μ*L [22]. A small sample volume was used in Rong Shao's LC-MS/MS method, but a concentration step was still needed after protein precipitation [19, 23–25]. In addition, hemolysis may occur during blood collection and processing, and hyperlipidemic samples are not rare in clinical sampling, especially in critically ill patients. The presence of hemolyzed or hyperlipidemic plasma samples may affect the analyte recovery efficiency [26–29]. In addition, the matrix effect of TGC in such abnormal plasma has not yet been reported.

The current research established a sensitive, simple, and rapid LC-MS/MS method for the quantitative analysis of TGC in patient plasma, utilizing tigecycline-d9 (TGC-d9) as an internal standard (IS). The influence of abnormal plasma on the matrix effect of TGC was investigated in the full method validation. This approach was applied to analyze 222 samples collected from 74 ICU patients for a population pharmacokinetic (PPK) study.

## 2. Materials and Methods

### 2.1. Chemicals and Reagents

Tigecycline (Lot: L290P45, 99% purity) was obtained from Beijing Bailingwei Technology Company. [t-Butyl-d9]-tigecycline (internal standard, IS, Lot: 0532724, 95% purity) was purchased from Cayman Chemical Company. High-pressure liquid chromatography- (HPLC-) grade formic acid was acquired from Sigma-Aldrich (St. Louis, MO, USA), and HPLC-grade acetonitrile was bought from Thermo Fisher Scientific (Waltham, MA, USA). Preparation of the ultrapure water was accomplished through a Milli-Q Plus water purification system.

### 2.2. Instrumentation

The HPLC system comprises of an LC-20AB pump, a CTO-20A column oven, and an SIL-20AC/HT autosampler (Shimadzu Corporation, Japan) coupled with a 4000Qtrap triple quadrupole mass spectrometer with a heated electrospray ionization source (Applied Biosystems, Foster City, CA, USA). Collection and processing of data was accomplished with Analyst 1.4.2 software.

### 2.3. LC-MS/MS Conditions

#### 2.3.1. Chromatographic Conditions

On an HSS T3 column (2.1 × 100 mm, 3.5 *μ*m particles) (Waters Company), the analyte and IS were separated by isocratic elution at a flow rate of 0.3 mL/min. The mobile phase was composed of water consisting of 0.1% formic acid (v/v) with 5 mM ammonium acetate (80% A) and acetonitrile (20% B). The needle rinsing solution of the autosampler was methanol : ultrapure water (v/v, 70 : 30). The column oven was set at 30°C, and the total analysis time was only 2 min. The sample volume injected by the autosampler was 3 *μ*L.

#### 2.3.2. Mass Spectrometric Conditions

**Figure 1:**
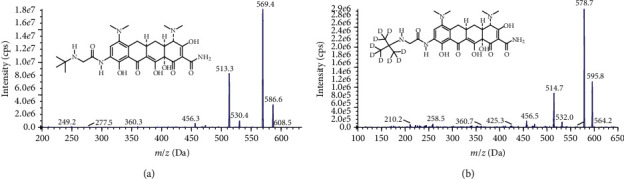
Chemical structures and product ion mass spectra of the [M + H]^+^ ions of (a) tigecycline and (b) [t-butyl-d9]-tigecycline (IS).

The mass spectrometer, scanning in multireaction monitoring (MRM) mode and using an electrospray ion source (ESI), operated in positive ion mode. The ion pairs used for quantitative analysis were *m*/*z* 586.4 ⟶ 513.3 for TGC (Figure 1(a)) and *m*/*z* 595.5 ⟶ 514.3 for the IS (Figure 1(b)). The positive electrospray ionization source (ESI+) temperature was 550°C; the ion spray voltage was 4500 V; and the declustering potential, entrance potential, and collision energies were 52 eV, 10 eV, and 20 eV for TGC and 70 eV, 10 eV, and 42 eV for the IS, respectively.

### 2.4. Preparation of Stock and Working Solutions

To prepare a tigecycline stock solution with a mass concentration of 1 mg/ml, 20.02 mg of tigecycline standard compound was weighed into a brown volumetric flask and diluted with 20 ml of ultrapure water. Calibration curves were obtained for the working solutions of tigecycline diluted with ultrapure water to obtain concentrations of 1000, 2000, 4000, 10000, 20000, 40000, 80000, and 100000 ng/ml. One milliliter of ultrapure water was added to the 0.5 mg internal standard to make an internal standard stock solution with a mass concentration of 500 *μ*g/ml. Five milliliters of water was added to 10 *μ*l of the internal standard stock solution to prepare a 1000 ng/ml internal standard working solution. All the stock working solutions were stored in a refrigerator at −80°C and thawed and mixed at room temperature before use.

### 2.5. Preparation of Standard and Quality Control Samples

Standard curves and quality control (QC) samples were prepared as follows: 10 *μ*L of each working solution was added to 190 *μ*L of blank plasma to prepare standard samples with final concentrations of 50, 100, 200, 500, 1000, 2000, 4000, and 5000 ng/mL. A similar method was used to acquire QC samples with plasma concentrations of 150, 750, 2250, and 3800 ng/ml. These samples were kept at −80°C until use.

### 2.6. Sample Preparation

Patient plasma samples were thawed at room temperature. To precipitate the protein in the plasma samples, a 50 *μ*l aliquot of the plasma samples was added to a 1.5 mL Eppendorf tube, mixed with a 20 *μ*l IS working solution aliquot, and vortexed for 20 seconds. Subsequently, 200 *μ*l of acetonitrile was added into the solution, followed by vigorous mixing for 1 minute and centrifugation at 12000 rpm at 4°C for 15 minutes. Three microliters of the supernatant was injected into the HPLC system.

### 2.7. Method Validation

Validation of the method was consistent with the guidance of the Fourth Edition of the Chinese Pharmacopoeia 2015, 9012 “Guidelines for Validation of Quantitative Analysis Methods for Biological Samples,” including the selectivity, carryover effects, lower limit of quantification (LLOQ), linearity, accuracy and precision, matrix effect, and stability.

#### 2.7.1. Selectivity and Carryover Effect

At least, six blank plasma samples collected from different individuals should be analyzed to test the selectivity of the method. The interference effect of each blank sample was determined by examining the peak area of retention times of TGC and the IS. When the response of the interfering component was less than 20% of the LLOQ and 5% of the internal standard, there was no interference under the verification criteria.

According to carryover criteria, the carryover effects should be evaluated by injecting blank samples after the upper limit of quantification (ULOQ) sample. Carryover in blank samples after the ULOQ sample should not exceed 20% of the LLOQ and should not exceed 5% of the internal standard.

#### 2.7.2. LLOQ and Linearity

LLOQ is the lowest concentration of analyte in a sample that can be reliably quantified with receivable accuracy and precision. Eight calibration standards were researched over a calibration range of 50–5000 ng/mL TGC in plasma to assess the linearity of the measurement. Each calibration curve was composed of eight calibration concentration levels and a double blank sample, a zero sample. A zero sample is a blank sample with only internal standard added. Linearity was determined by fitting the peak area ratio to the analyte concentration using weighted least squares analysis and a weighting factor of 1/*x*^2^. In method validation, at least, 3 calibration curves should be evaluated. The back-calculated concentration of the calibration standard should generally be within ±15% of the labeled value, and the LLOQ should be within ±20%. At least, 75% calibration standards, containing, at least, 6 effective concentrations, should meet the abovementioned standards. If the result of a calibration standard sample does not meet these standards, the standard sample should be rejected. The calibration curve that does not contain this standard sample should be re-evaluated.

#### 2.7.3. Accuracy and Precision

Interbatch and intrabatch accuracy and precision were assessed by evaluating six replicate QC samples at LLOQ, low QC (LQC1), low-medium QC (LQC2), medium QC (MQC), and high QC (HQC)concentrations (50, 150, 750, 2250, and 3800 ng/mL) in, at least, three batches performed over, at least, two days. The accuracy calculated as the percent deviation from the nominal concentration describes how close the measured value is to the nominal concentration of the analyte. Precision is determined by the coefficient variation (CV%) of the calculated vs. nominal concentration. The average accuracy should generally be within ±15% of the labeled value of the quality control sample, and the accuracy of the LLOQ should be within ±20% of the labeled value. For quality control samples, CV% between batches and within batches should generally not be more than 15%, and the coefficient of variation obtained for the LLOQ should not exceed 20%.

#### 2.7.4. Matrix Effect

Since the accuracy of the LC‐MS/MS experiment might be affected by matrix effects, we then investigated the matrix effects by examining the blank matrix collected from six individuals at three QC levels (150, 750, and 3800 ng/mL). The matrix factor of an analyte and IS should be computed by determining the ratio of the peak area with the matrix (quantified by adding the analyte and IS after blank matrix extraction) to the corresponding peak area short of the matrix (the pure solution of the analyte and IS). The IS-normalized matrix factor (MF) was obtained by determining the ratios of the MF values of the analyte and the IS. The CV% of the IS-normalized MF computed from six batches of the matrix should not be greater than 15%.

In addition to the normal matrix effect, attention should also be paid to the matrix effects of other samples, such as hemolyzed samples and hyperlipidemic plasma samples. To investigate the effect of the hemolysis and hyperlipidemic status on quantitative detection, simulated hemolytic plasma containing 2% lysed blood (2% hemolytic plasma) was prepared by adding 20 *μ*L of whole blood subjected to multiple freeze-thaw cycles to 980 *μ*L of normal blank plasma. Hyperlipidemic plasma was mimicked by adding 15 *μ*L of a 20% medium-/long-chain fatty acid emulsion to 985 *μ*L of normal blank plasma to obtain a final triglyceride content of approximately 1.7 mM.

#### 2.7.5. Stability

Using three replicate samples with low and high QC levels, we assessed the stability of TGC in plasma under various storage and processing conditions. We evaluated the stability of TGC in human plasma in the short-time by incubating QC samples at room temperature and storing at 2–8°C for 24 hours. The long-term stability of QC samples stored at −80°C for 130 days was determined. For evaluation of postpreparation, QC samples were collected before injection and placed in the autosampler for 30 h at 30°C. The freeze-thaw stability was determining after five complete freeze-thaw cycles (−80°C to room temperature) were performed. If the measured value is within the acceptable accuracy (±15%) and CV% (≤15%) range, these samples are considered stable.

### 2.8. Application to the PPK Study

The verified method was effectively applied to a PPK study of 74 patients in two ICU datasets. The study was approved by the Research Ethics Committee of Guangdong Provincial People's Hospital (Guangdong Academy of Medical Science) (approval no. GDREC2018268H (R1)). Three plasma samples were collected from each patient after the blood concentration reached steady state, usually after the fifth dose. The collection times were at the end of the installation, 4–6 hours after intravenous administration, and before the next dose. All blood samples were collected into EDTA-K2 tubes and immediately centrifuged at 3,000 rpm for 10 min; the resultant plasma samples were stored and frozen at −80°C until analysis. A total of 222 plasma samples were collected and analyzed.

## 3. Results and Discussion

### 3.1. Method Optimization

The mass spectrometer, scanning in multireaction monitoring (MRM) mode and using an electrospray ion source (ESI), operated in positive ion mode. The ion pairs used for quantitative analysis were *m*/*z* 586.4 ⟶ 513.3 and *m*/*z* 595.5 ⟶ 514.3 for TGC and the IS, respectively (Figure 1). TGC is sensitive to the pH of the mobile phase; therefore, to obtain better chromatograms, a buffer containing 0.1% formic acid (v/v) with 5 mM ammonium acetate was used as mobile phase A. The total run time was only 2 min, which is much shorter than the run times of previously published methods [19, 22].

### 3.2. Method Validation

#### 3.2.1. Selectivity and Carryover Effect

**Figure 2:**
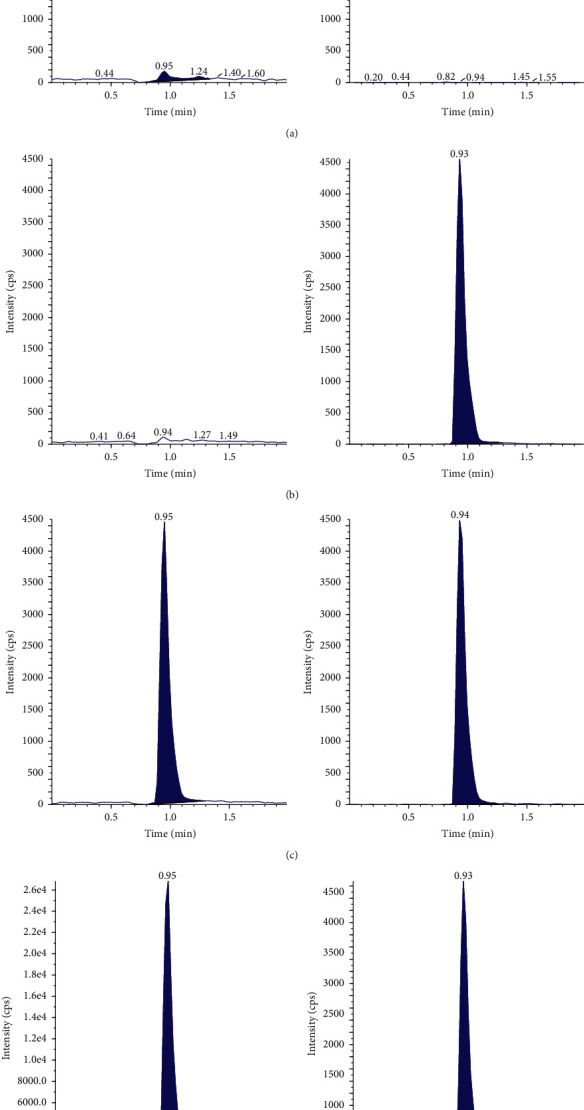
The chromatograms of TGC and IS in human plasma. Left is TGC, and right is IS. (a) Double-blank plasma sample; (b) blank plasma sample; (c) lower limit of quantification (LLOQ) plasma samples; and (d) a patient's plasma sample.

The retention times of TGC and the IS were 0.95 min and 0.94 min, respectively. As indicated, the interference peaks of endogenous substances observed at the retention time of TGC and the IS were negligible (Figure 2), which implies that the MRM chromatograms are typical of (a) double-blank plasma, (b) blank plasma spiked only with IS, (c) TGC (LLOQ) and IS spiked with blank plasma, and (d) plasma from a patient. This confirmed the specificity of the developed method for the quantitative analysis of TGC in human plasma.

By adding a double blank sample after the ULOQ sample, the observed peak area is less than 20% of the LLOQ and 5% of the IS, indicating that there is no carry over of the analyte and IS existed in the method.

#### 3.2.2. The LLOQ and Linearity

LLOQ is 50 ng/mL. A typical linear regression equation is *y* = 15.7*x* + 0.0498 (*r* = 0.9978), where *y* represents the ratios of the TGC peak area to the IS peak area and *x* stands for the concentrations of TGC. The peak area ratios and concentration showed a linear relationship in the range of 50–5000 ng/mL, with correlation coefficients >0.99. The slopes and correlation coefficients were consistent between batches. A linear range of 50–5000 ng/mL was adequate for the PK estimates of TGC in ICU patients receiving standard or high-dose regimens because almost all TGC concentrations were in this range. A higher ULOQ was needed for the application of a high-dose regimen, in which concentrations above 3000 ng/mL might be observed in the elimination phase of TGC [4].

#### 3.2.3. Accuracy and Precision

The intraday precision (CV%) ranged from 1.7 to 3.6% over the five concentration levels of QC samples, and the accuracy was within 93.8 to 101.8%. For the interday experiments, the precision varied from 3.7 to 7.2%, and the accuracy was within 93.4 to 99.7% at these levels. The results of accuracy and precision analyses are summarized in Table 1. A low LLOQ needed additional sample volume or a concentration process after protein precipitation, as reported [19, 22], but no concentration lower than the LLOQ was found in the present study. An extremely low LLOQ was unnecessary in the TDM of TGC.

#### 3.2.4. Matrix Effect

The CV% of the IS-normalized matrix effect was within the range from 4% to 8.5%, which is in accordance with the guidelines (Table 2). More importantly, we found that the IS-normalized matrix effect of TGC in the hemolytic and hyperlipidemic plasma was similar to that in normal plasma. Therefore, our method has good potential for clinical applications of TDM in ICU patients.

#### 3.2.5. Stability

Table 3 shows the short‐term, long‐term, freeze-thaw (five cycles), and autosampler stabilities of TGC in plasma. Plasma samples can be stable for 24 h at room temperature or at 2–8°C. The postpreparative samples were also stable in the autosampler maintained at 30°C for, at least, 30 h. TGC was found to be stable for a maximum of five freeze and thaw cycles. Only three freeze and thaw cycles were reported in previous studies [19, 20]. The long-term stability results showed that TGC was stable in human plasma for up to 130 days at −80°C.

### 3.3. Application

Severely ill patients have complex physiologies, often have liver and kidney dysfunction, and have various infections; therefore, there are large differences between their metabolisms and drug clearance. Therefore, it is necessary to detect the blood concentration to determine whether the conventional dose can achieve an effective treatment rate. In this study, we collected 222 samples and tested the TGC concentration in patients who received tigecycline to treat lung infections in ICUs. The peak concentration after dose (*C*_max)_ of TGC is 871.5 ± 467.3 ng/ml, the concentration within 6 hours after administration is 334.8 ± 220.3 ng/ml, and the trough concentration (*C*_min_) of TGC is 260.9 ± 190.1 ng/ml.

## 4. Conclusions

The established method exhibited good specificity, precision, accuracy, and linearity in the range of 50–5000 ng/ml. Only 50 *μ*L of plasma is required, and the supernatant used for detection can be obtained by centrifuging plasma that was processed by a one-step protein precipitation method, which is quite convenient, practical, and fast. Compared with the method reported in the literature, our method has a detection time of only 2 minutes. The established method is suitable for the analysis of clinical plasma samples, including hemolyzed or hyperlipidemic samples, and was successfully applied to a PPK study of 74 ICU patients. This model can be used to estimate TGC exposure in future PK/PD studies on the efficacy and safety of TGC administered to ICU patients.

## Figures and Tables

**Table 1:** The precision and accuracy of the determination of TGC in human plasma (*n* = 6).

Nominal concentration (ng/ml)	Intrabatch (*n* = 6)			Interbatch (*n* = 18)		
	Measured concentration (ng/ml, mean ± SD)	CV%	Accuracy%	Measured concentration (ng/ml, mean ± SD)	CV%	Accuracy%
50	49 ± 1.8	3.6	98.6	47 ± 3.3	7.2	93.4
150	153 ± 2.7	1.8	101.8	150 ± 5.5	3.7	99.7
750	730 ± 12.7	1.7	97.3	716 ± 30.2	4.2	95.4
2250	2277 ± 48.0	2.1	101.3	2206 ± 112.8	5.1	98.1
3800	3565 ± 105.4	3.0	93.8	3739 ± 214.2	5.7	98.4

**Table 2:** Matrix effect of TGC in various plasma matrices.

Nominal concentration (ng/ml)	Normalized plasma		Hemolyzed plasma		Hyperlipidemic plasma	
	Matrix effects (%)	IS-normalized matrix effects (%)	Matrix effects (%)	IS-normalized matrix effects (%)	Matrix effects (%)	IS-normalized matrix effects (%)
150	209 ± 11.0	103 ± 6.9	209 ± 1.4	104 ± 5.6	200 ± 5.2	95 ± 3.2
750	182 ± 1.9	87 ± 5.2	193 ± 9.3	96 ± 6.0	179 ± 1.4	93 ± 3.8
3800	149 ± 1.6	95 ± 2.0	152 ± 3.4	95 ± 2.1	150 ± 1.5	98 ± 2.1
CV (%)	14.5	8.5	13.8	6.0	12.0	4.0

**Table 3:** Stability of TGC in human plasma under tested conditions.

Stability	Nominal concentration (ng/mL)	Stability sample concentration (ng/mL)	Accuracy (%)	CV (%)
Short‐term (24 h, 25°C)	150	168 ± 7.0	112.3	4.2
	3800	3753 ± 206.5	98.8	5.5

Short‐term (24 h, 4°C)	150	158 ± 14.7	105.4	9.3
	3800	3673 ± 212.0	96.8	5.8

Freeze-thaw (five cycles, −80−25°C)	150	150 ± 3.8	100.1	2.5
	3800	3993 ± 293.6	105.1	7.4

Long‐term (130 days, −80°C)	150	156 ± 11.1	103.8	7.1
	3800	3524 ± 97.4	92.7	2.8

Autosampler (30 h, 30°C)	150	162 ± 10.4	108.2	6.4
	3800	4021 ± 269.0	105.8	6.7

## Data Availability

The data used to support the findings of this study are included within the article.

## Abbreviations

IS: Internal standard
LC-MS/MS: Liquid chromatography-tandem mass spectrometry
TGC: Tigecycline
MRM: Multireaction monitoring
ESI: Electrospray ion source
FDA: Food and Drug Administration
AUC24: The area under the concentration-time curve from 0 to 24 h at steady state
MIC: Minimal inhibitory concentration
CL: Clearance
TDM: Therapeutic drug monitoring
PK/PD: Pharmacokinetic/pharmacodynamic
UV: Ultraviolet
TGC-d9: Tigecycline-d9
ICU: Intensive care unit
PPK: Population pharmacokinetic
HPLC: High-pressure liquid chromatography
QC: Quality control
LLOQ: Lower limit of quantification
ULOQ: Upper limit of quantification
CV%: Coefficient variation
MF: Matrix factor
*C*_max_: The peak concentration after dose
*C*_min_: The trough concentration.
